# The Legacy of Sexual Ancestors in Phenotypic Variability, Gene Expression, and Homoeolog Regulation of Asexual Hybrids and Polyploids

**DOI:** 10.1093/molbev/msz114

**Published:** 2019-05-11

**Authors:** Oldřich Bartoš, Jan Röslein, Jan Kotusz, Jan Paces, Ladislav Pekárik, Miloslav Petrtýl, Karel Halačka, Eva Štefková Kašparová, Jan Mendel, Alicja Boroń, Dorota Juchno, Anna Leska, Olga Jablonska, Vladimir Benes, Monika Šídová, Karel Janko

**Affiliations:** 1 Institute of Animal Physiology and Genetics, Laboratory of Fish Genetics, The Czech Academy of Sciences, Libechov, Czech Republic; 2 Department of Zoology, Faculty of Science, Charles University, Prague, Czech Republic; 3 Department of Biology and Ecology, Faculty of Science, University of Ostrava, Ostrava, Czech Republic; 4 Museum of Natural History, University of Wroclaw, Wroclaw, Poland; 5 Institute of Molecular Genetics, Laboratory of Genomics and Bioinformatics, The Czech Academy of Sciences, Prague, Czech Republic; 6 Plant Science and Biodiversity Center, Institute of Botany, Slovak Academy of Sciences, Bratislava, Slovakia; 7 Faculty of Education, Trnava University, Trnava, Slovakia; 8 Department of Zoology and Fisheries, Faculty of Agrobiology, Food and Natural Resources, Czech University of Life Sciences Prague, Prague, Czech Republic; 9 Institute of Vertebrate Biology, Czech Academy of Sciences, Brno, Czech Republic; 10 Department of Zoology, Faculty of Biology and Biotechnology, University of Warmia and Mazury in Olsztyn, Olsztyn, Poland; 11 Genomics Core Facility, European Molecular Biology Laboratory (EMBL), Heidelberg, Germany; 12 Institute of Biotechnology of the Czech Academy of Sciences – BIOCEV, Vestec, Czech Republic

**Keywords:** asexuality, polyploidy, hybridization, tissue-specific gene expression, expression-level dominance, cis-/trans-regulation

## Abstract

Hybridization and polyploidization are important evolutionary processes whose impacts range from the alteration of gene expression and phenotypic variation to the triggering of asexual reproduction. We investigated fishes of the *Cobitis taenia-elongatoides* hybrid complex, which allowed us to disentangle the direct effects of both processes, due to the co-occurrence of parental species with their diploid and triploid hybrids. Employing morphological, ecological, and RNAseq approaches, we investigated the molecular determinants of hybrid and polyploid forms.

In contrast with other studies, hybridization and polyploidy induced relatively very little transgressivity. Instead, *Cobitis* hybrids appeared intermediate with a clear effect of genomic dosing when triploids expressed higher similarity to the parent contributing two genome sets. This dosage effect was symmetric in the germline (oocyte gene expression), interestingly though, we observed an overall bias toward *C. taenia* in somatic tissues and traits. At the level of individual genes, expression-level dominance vastly prevailed over additivity or transgressivity. Also, trans-regulation of gene expression was less efficient in diploid hybrids than in triploids, where the expression modulation of homoeologs derived from the “haploid” parent was stronger than those derived from the “diploid” parent.

Our findings suggest that the apparent intermediacy of hybrid phenotypes results from the combination of individual genes with dominant expression rather than from simple additivity. The efficiency of cross-talk between trans-regulatory elements further appears dosage dependent. Important effects of polyploidization may thus stem from changes in relative concentrations of trans-regulatory elements and their binding sites between hybridizing genomes. Links between gene regulation and asexuality are discussed.

## Introduction

Interspecific hybridization and polyploidization are phenomena with great evolutionary importance. Genome merging into allodiploid or allopolyploid individuals can directly affect the formation of new species either by direct creation of new lineages or by establishment of reproductive barriers in the case of unfit hybrids (e.g., Russell 2003; Bell and Travis 2005) and hybridization can result in a wide range of phenotypic outcomes. At one extreme, hybrids may appear as intermediate forms whose variability is embedded within the limits determined by parental species. At the other extreme, hybridization may induce novel forms whose traits values are higher or lower than those observed in the original parents, that is, transgressive trait expression (Bell and Travis 2005). The addition of extra genomes during polyploidization further modifies the dosage of hybridizing genomes, which is expected to affect the ratio of their transcription products. Such a change is particularly likely to have complex consequences for the stoichiometry of protein components of macromolecular complexes and may ultimately affect the expression of traits (Kearney et al. 2004; Kierzkowski et al. 2011; Birchler and Veitia 2012). Yet, despite recent progress in NGS technologies, many uncertainties around the molecular processes underlying such a variety of phenotypic outcomes still remain (Deans et al. 2015). Even straightforward patterns may be generated by very different mechanisms; for example, hybrids with apparently intermediate phenotypes may form even if majority of their genes is nonadditively expressed (rev. in e.g., Yoo et al. 2014; Fridman 2015). Therefore, there has been intense debate over whether there are some common rules underlying the variety of outcomes, or whether observed data result from rather case-specific effects.

The amount of accumulated genetic divergence between hybridizing genomes is a candidate parameter that may systematically impact on hybrids’ or polyploids’ phenotypes. That interparental divergence affects the quality of hybrids’ phenotypes has become clear since Bateson’s (1909), Dobzhansky’s (1959), and Muller’s (1942) models of accumulating reproductive incompatibilities (BDMI models) and it may also affect the likelihood of establishment of allopolyploids (Chapman and Burke 2007; Paun et al. 2009 but see Buggs et al. 2009). Less clear, however, is how such divergence could mechanistically translate into hybrid phenotypes. For example, some studies suggest the appearance of hybrid abnormalities may be due to divergence in noncoding DNA (e.g., Greig 2009; Balcova et al. 2016), thereby matching Bateson’s view, whereas others point to altered interactions among genes, which is closer to Dobzhansky’s and Muller’s genic models (Forsdyke 2011).

Comparative analyses of diverse hybrid and/or polyploid phenotypes are often performed to disentangle general rules from case-specific effects. However, there is one specific outcome of hybridization that has traditionally been investigated separately—that is, the frequent shift in hybrids’ reproduction toward asexuality. Interestingly, the idea that the likelihood of asexuality correlates with the interparental genetic divergence was formulated just a few years after Bateson’s speciation model by Ernst (1918). Much later, Moritz et al. (1989) formulated “balance hypothesis” (BH), which predicts that hybrid asexuality can arise only in a specific interval when the genomes of parental species are divergent enough to disrupt meiosis, yet not divergent enough to seriously compromise hybrid viability or fertility. The BH also aids in understanding the strong link between asexuality and polyploidy. Indeed, it has been documented that asexual hybrids between any two species generally exist in only one ploidy level (usually successful clones originating from hybridization between a pair of species are either diploid or triploid but not both; rev. in Choleva and Janko [2013]). Because BH posits that the production of unreduced gametes in hybrids depends on properly balanced genomic dosage, Moritz et al. (1989) proposed that incorporation of an extra genome must affect the fecundity and viability of resulting allopolyploids by shifting the dosage of transcripts of hybridizing genomes. Such shifts may thus either stabilize the original diploid lineage, which produces a high proportion of unreduced gametes with low viability, or destabilize a successful diploid clone by disrupting its genetic balance between allelic products ensuring clonality.

Recently, Janko et al. (2018) proposed that models describing the relationship between hybridization and asexuality as well as those explaining the BDMI may be viewed as two sides of the same coin because hybrid asexuality arises as an inherent stage of the speciation process itself. This is because nascent species tend to produce fertile sexual hybrids at initial stages of differentiation, but the likelihood of asexual reproduction rises with increasing divergence of hybridizing genomes. At the same time, asexual gametogenesis in hybrids hampers interspecific gene exchange and therefore appears as a special case of postzygotic barrier that tends to evolve earlier during species divergence than other barriers such as sterility or lethality. However, as with BDMI, it remains unclear how does the accumulating interparental divergence affect the distortion of the hybrids’ reproductive mode. Some researchers have focused on the role of decreasing sequence homology between chromosomes (De Storme and Mason 2014), whereas others have focused on the accumulation of epistatic interactions among genes that disrupt meiosis (Moritz et al. 1989). Alternatively, Carman (1997) suggested that rather than being a mere consequence of accumulated divergence, it is the asynchronous expression of differently timed developmental programs brought together by hybridization that ultimately distorts hybrid’s gametogenesis toward the production of unreduced gametes.

It is becoming increasingly appreciated that many effects of genome merging are due to divergence in cis-/trans-regulatory elements that modify homoeolog regulation in hybrids (Tulchinsky et al. 2014). Although cross-talk between transcription factors and their binding sites originating from both parents is possible when divergence of their genomes is low, it becomes hampered when divergence is high, therefore promoting the regulation of homoeologs by their own genome-specific signals (rev. in Yoo et al. [2014]). We think that the link between genetic divergence and the cis-/trans-regulation may also be important for the evolution of hybrid asexuality since Carman’s (1997) model postulates that asynchronous expression of both developmental programs in a hybrid may only occur when alleles of the key genes are regulated independently by their own genome-specific signals. Therefore, although not stated explicitly, Carman’s model implies pervasive cis-regulation or at least inefficient cross-talk between parental copies of trans-elements.

To understand the effects of hybridization and polyploidy on phenotypes and reproductive modes, it is therefore necessary to investigate gene expression modulation. Although relatively few studies have compared gene expression patterns in related sexual and asexual forms (e.g., Hanson et al. 2013; Li et al. 2014; Matos et al. 2015), hybrid and polyploid organisms are intensively studied in general. Unfortunately, disentangling the direct impacts of hybridization and polyploidization is difficult since both phenomena are closely linked (i.e., many taxa are either allodiploid or allopolyploid, but not both; Kearney 2005; Lundmark 2006). Consequently, relatively few studies have succeeded in separating both effects (e.g., Hegarty et al. 2006; Buggs et al. 2011; Xu et al. 2014). Moreover, investigated polyploid organisms often stem from rather ancient polyploidization events suggesting that their regulatory networks have surely been modified by many postformational changes (Gianinetti 2013) and the absence of the original parental taxa can prevent effective comparisons to be made (Yoo et al. 2014).

This study aims at disentangling the direct effects of hybridization and polyploidy on gene expression modulation, morphological variation, and ecological characteristics of diploid and polyploid hybrids. We focus on a model system of hybrids between different species of European spined loaches (*Cobitis*, *Teleostei*), which are particularly well suited for such purposes as parental species simultaneously co-occur with hybrids of two ploidy levels (diploid and triploid). Moreover, this allows us to investigate the effects of gene regulation on aberrant reproductive modes because hybridization of *Cobitis* directly induces clonality (Choleva et al. 2012). Specifically, we focused on two parental species, *C. elongatoides* (2*n* = 50 chromosomes) and *C. taenia* (2*n* = 48) (hereafter also called as EE and TT, respectively, where E and T denote haploid genome), which diverged about 9 Mya (Janko et al. 2018) and were initially interconnected by intensive gene exchange. With ongoing divergence, these species lost the capacity to produce sexual hybrids (Janko et al. 2018). Currently, reproductive contact between the two species in Central Europe leads to diploid hybrids (hereafter ET), which are either sterile males or clonal females (Janko et al. 2005; Janko, Flajšhans, et al. 2007). Clonal reproduction is achieved by gynogenesis when the development of unreduced oocytes is triggered by a sperm from a sexual species, whose genome is generally degraded afterward. Still, about one-third of clonal eggs become fertilized, resulting in triploid progeny with either EET or ETT genomic constitution. They also reproduce gynogenetically (Janko, Bohlen, et al. 2007; Choleva et al. 2012; Janko et al. 2018) and occasionally produced tetraploid progeny are generally inviable (Janko, Bohlen, et al. 2007; Juchno et al. 2014). Natural clonal lineages transmit the parental genomes en block for thousands of generations without notable chromosomal rearrangements (Majtánová et al. 2016). Available data indicated substantial phenotypic overlap between parental, hybrid and polyploid forms but some apparently transgressive traits have been detected. Specifically, hybrids differ in several morphological and ecological traits from both parents (Kotusz 2008; Kotusz et al. 2014; Fedorčák, Pekárik, et al. 2017), furthermore polyploids have larger cells than diploid biotypes resulting in lower standard metabolic rates (Maciak et al. 2011) and probably also growth rates (Fedorčák, Koščo, et al. 2017).

Here, we investigate the correlation between the plasticity of external phenotypic traits correlate and underlying gene expression and test whether differences between parental species and their hybrids result from additive, transgressive or dominant expression of genes and traits. We also address the underlying gene expression modulation, specifically the regulation via cis- and trans-interactions. To achieve these aims, we compared at several levels the variability of traits in females belonging to both parental species as well as their di- and tri-ploid hybrids. Namely, we investigated their 1) habitat preferences as a proxy for biotype–environment interaction, 2) morphology as a proxy for biotype-specific ontogenetic development, 3) the mRNA expression profiles as a mean for understanding the gene expression modulation and inheritance of gene expression, and finally, 4) the allele-specific expression (ASE) as a proxy for cis- and trans-regulatory divergences and homoeolog expression modulation in hybrids. To get some insight into expression-regulation differences between somatic and germinal tissues, which may have some link to asexuality, we performed RNAseq on livers and oocytes.

## Results

### The Effects of Hybridization and Polyploidy on Differences among Biotypes in Morphology, Habitat Preferences, and Overall Gene Expression

**Fig. 1.**
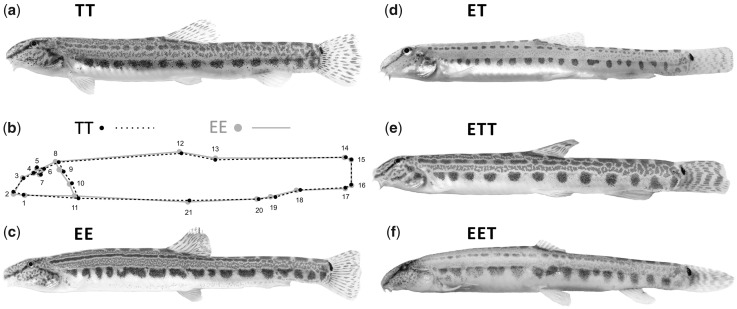
(*b*) Morphological difference between the parental species *Cobitis taenia* and *C. elongatoides* depicted by the translated positions of landmarks, an average shape of both groups (gray) and shape changes associated with PC2 (black). (*a*, *c*–*f*) The five biotypes included in this analysis are shown.

To begin with, we tested for pervasive differences among parental species, hybrids and polyploids in terms of phenotypic variability including morphology, habitat preferences, and gene expression. Morphological variability of 191 females of all biotypes was analyzed using geometrical morphometry. Despite extensive overlap in body shape among studied biotypes and similarity of both parental species in general habitus (fig. 1), the canonical variable analysis (CVA) with Jackknife cross-validation provided high overall classification accuracy 86.911% (Kappa = 0.810). Additionally, the Permutational Multivariate ANOVA (PERMANOVA) of procrustes coordinates found significant differences between all pairs of biotypes (*P* < 0.03 for all pairwise comparisons), altogether confirming the existence of biotype-specific differences in the body shape (fig. 1). These differences mainly concerned variable proportions of head robustness associated with different body length/height ratios, the relative positions of pectoral, ventral and anal fins, and the position of the anus. In general, *C. elongatoides* females had slightly deeper bodies with shorter heads and longer caudal peduncles than *C. taenia* (fig. 1*b*).

**Fig. 2.**
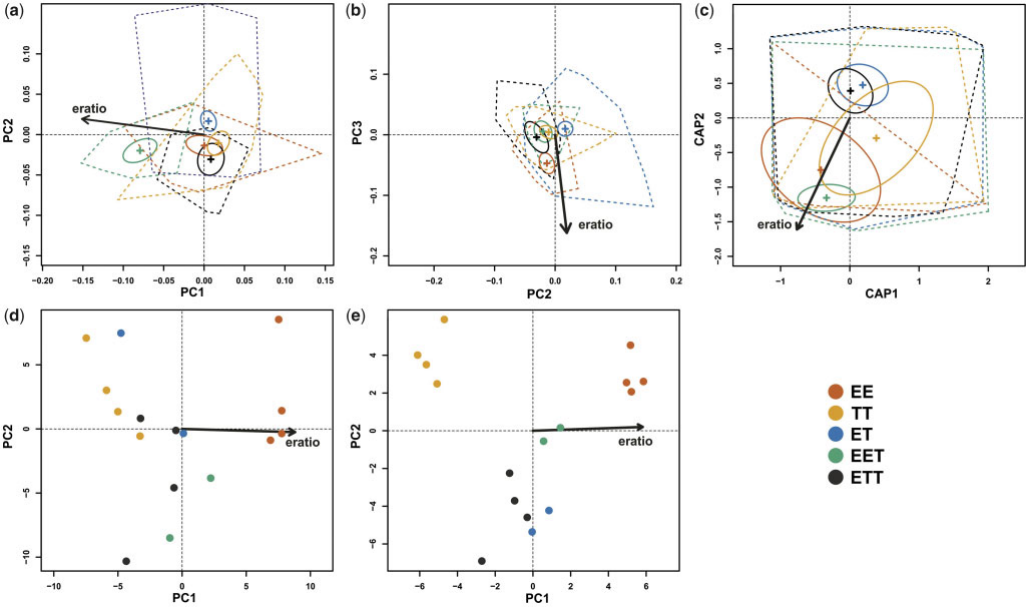
PCAs of morphological (*a*, *b*), habitat (*c*), RNAseq oocytes (*d*), and RNAseq livers (*e*) data. Individuals belonging to respective biotypes are marked by colors as indicated in the legend. Arrow labeled as “eratio” indicates the direction of fitted gradient defined by the proportion of *Cobitis elongatoides* genome in hybrid forms. Ellipses denote the confidence interval of the position of centroid for given biotype. Dotted shapes represent convex hulls enclosing positions of all individuals for given biotype. Explained variability of axes were in panels *a* and *b*: PC1 = 31.70%, PC2 = 22.46%, and PC3 = 11.27%; in panel *c*: PC1 = 44.21% and PC2 = 28.35; in panel *d*: PC1 = 28.84% and PC2 = 13.43%; and in panel *e*: PC1 = 23.07% and PC2 = 14.66%.

By contrast, we did not find strong differences in habitat preferences as all loach biotypes occupied generally similar microhabitats characterized by sandy-muddy bottom substratum and water velocity of about 0.1 m/s (fig. 2*c*). Consequently, CVA with Jackknife cross-validation provided low classification accuracy (45.771%, Kappa = 0.233). However, individual’s microhabitat usage was still significantly predicted by its biotype (PERMANOVA, DF = 4, *F* = 9.510, *R*^2^ = 0.163, *P* < 0.001) and significant differences were found between EE-ET, EE-ETT, EET-ET, EET-ETT, and TT-EET pairs (all *P* < 0.014). The fact that *C. elongatoides* and *C. taenia* do not co-occur at any site might have affected this analysis. Therefore, we tested whether observed patterns could reflect differences among sites inhabited by either of the parental species. Thus, we included the division of sites into *elongatoides*- and *taenia*-specific into PERMANOVA. However, the effect of site type was negligible (DF = 1, *F* = 1.845, *R*^2^ = 0.008 *P* = 0.139), whereas the effect of biotype was still highly significant (DF = 4, *F* = 9.550, *R*^2^ = 0.163, *P* < 0.001).

To investigate the gene expression differences between parental species and their hybrid biotypes, we used an RNAseq approach (sequencing results shown in supplementary table 1, Supplementary Material online). We tested for differentially expressed genes (DEGs) between groups that were a priori defined in a way that data could be partitioned according to three following factors: first, we contrasted two parental species against each other (i.e., EE vs. TT biotypes) to reveal DEGs between the species; next, we contrasted sexual and asexual individuals against each other (i.e., {EE, TT} vs. {ET, EET, ETT} biotypes) to reveal DEGs characterizing asexual hybrids and finally, we contrasted diploid and polyploid individuals against each other (i.e., {EE, TT, ET} vs. {EET, ETT} biotypes) to reveal DEGs associated with polyploidy.

**Fig. 3.**
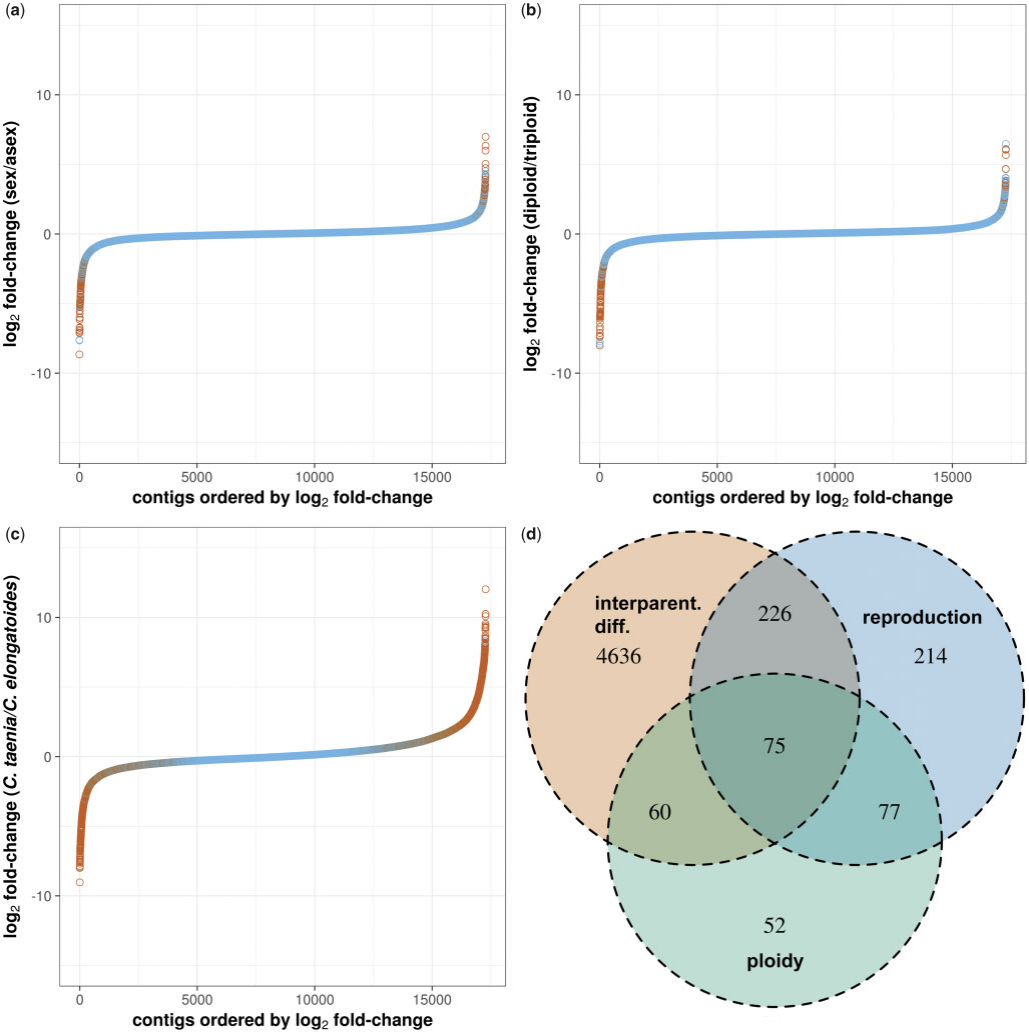
(*a*–*c*) Plots of ordered genes according to log_2_ FC between defined groups for oocyte tissue (red dots represent DEGs at FDR < 0.05). (*d*) Venn diagrams demonstrating the overlap between groups of DE genes found by ANOVA type II as significantly affected by some of the following factors: (1) interparental differentiation (assuming the individuals position along the *Cobitis elongatoides—*general hybrid*—C. taenia* continuum), (2) reproduction (sexual vs. asexual), and (3) ploidy (diploid vs. triploid).

In total, we found 4,787 of DEGs in liver tissue and 5,606 in oocytes, of which most were attributable to differences between both sexual species (i.e., 4,245 and 5,205 detected DEGs in livers and oocytes, respectively). Significantly more “interspecific” DEGs were up regulated in *C. taenia* than in *C. elongatoides* (i.e., 2,362 in livers and 2,921 in oocytes, binomial test, *P* < 10^−4^ in both tissues; fig. 3 for oocytes and supplementary fig. 1, Supplementary Material online, for livers). A relatively small proportion of DEGs (i.e., 514 genes in livers and 399 in oocytes, respectively) were transgressive with hybrids’ expression being higher or lower than that of both parental species. We acknowledge that power of such analysis may theoretically be diminished by the fact that two different parentals were treated as one group with consequently artificially increased variance. However, a similarly low proportion of transgressive genes were revealed using the categorization sensu (Yoo et al. 2013; see below), where parental species were treated separately, thereby corroborating the interpretation that hybridization did not introduce drastic changes. Even less genes (i.e., 408 in livers and 232 in oocytes, respectively) were significantly affected by ploidy. Both these “asexual” and “polyploidy” DEGs had asymmetric distribution with more common upregulation (ca. 60% of DEGs were upregulated in both tissues of asexuals, whereas in polyploids there were ∼80% and ∼70% of DEGs upregulated in oocytes and livers, respectively; see fig. 3 for oocytes and supplementary fig. 1, Supplementary Material online, for livers).

ANOVA type II was employed to test for potential interactions between the aforementioned factors, which involved differentiation between parental species, between sexual and asexual forms, and between diploids and polyploids (see Materials and Methods for details). It showed that most genes, which differed between parental species were not simultaneously significantly affected by hybridization and/or polyploidy. In contrast, more than half of “asexual” and “polyploidy” DEGs were also significantly affected by underlying expression differentiation between parental species (fig. 3*d* for oocytes and supplementary fig. 1*d*, Supplementary Material online, for livers). This suggests that part of those “asexual”- and “polyploidy”-specific genes does not necessarily indicate transgressive expression, but instead their expression follows the interspecific gradient. In other words, hybrids may appear unique not just because their expression levels transgress parental levels but also because they are intermediate between two already differentiated species. The results of the Gene Ontology (GO) terms enrichment analysis are summarized in supplementary table 2, Supplementary Material online.

In summary, all morphological, environmental, and transcriptomic data point to the fact that the greatest differences exist between both parental species, but diploid and polyploid hybrids also possess some unique characters.

### The Effect of Genomic Dose on among Biotype Differences in Morphology, Habitat Preferences, and Overall Gene Expression

Subsequently, we evaluated whether and to what extent the overall dis/similarity of hybrids to either one or the other parent depends on the dose of parental genomes. To do so, we evaluated the effect of the genomic dose on the expression of morphological and RNA data using Principal Component Analysis (PCA) and of ecological traits using Principal Coordinates Analysis (PCoA).

The Broken-stick model indicated the significance of the first three axes in PCA of morphological data (fig. 2*a* and *b*) where relative positions of individuals along these axes reflected a vector loaded with natural (body shape) and artificial sources of variability. Despite great care to present the fish as straight as possible, post mortem deviations from the horizontal position still represent an issue, especially in fishes with elongated body (Marcil et al. 2006; Fruciano 2016). Consequently, several statistical corrections were suggested to cope with artificial variation attributable to such body arching (i.e., Burnaby 1966; Valentin et al. 2008; Fruciano 2016), but these have also been criticized (Angielczyk et al. 2004). In our data, the majority of arching effect was concentrated on PC1, whereas PC2 mostly reflected the differences between di- and tri-ploids and PC3 tended to discriminate between both parental species with hybrids occupying intermediate positions. Therefore, we discarded PC1 from further analyses and tested whether individual’s position in the ordination biplot of axes PC2 and PC3 is correlated with its characteristic proportion of E-genome (e.g., pure *C. elongatoides* [EE] = 100%, EET = 66.6%, ET = 50%, and ETT = 33.3% and pure *C. taenia* [TT] = 0%). We found that an individual’s genomic composition explained a relatively small but significant (∼6%) part of the total morphological variability (fig. 2; *R*^2^ = 0.063, *P* < 0.003) and that it had a significant effect on an individual’s position in morphospace, thereby demonstrating that body shapes are affected by genomic dose. However, the gradient of phenotypic similarity of hybrids to parental species was not simply proportional to the ratio of parental genomes composition within the hybrids; although triploids appeared closer to the parental species contributing two chromosomal sets, diploids were not fully intermediate and all hybrid forms were generally more similar to *C. taenia* than to *C. elongatoides* (fig. 2 and supplementary fig. 2*b*, Supplementary Material online).

The first four axes in PCoA of microhabitat data were significant according to the Broken-stick model and the strongest correlation between an individual’s E-genome proportion and position in the multivariate space was found in the ordination biplot of axes 1 and 2 (fig. 2). Again, an individual’s genomic composition significantly affected its position in the biplot and explained over 10% of variance (*R*^2^ = 0.110, *P* < 0.001). As with morphology, the gradient was not linear since diploid hybrids were again more similar to *C. taenia* rather than being fully intermediate (fig. 2 and supplementary fig. 2*a*, Supplementary Material online).

The Broken-stick model indicated that only first axis was significant on PCA of both oocyte and liver RNAseq data. We revealed very strong effect of genomic dosage (denoted as E-genome proportion) on gene expression patterns in both tissues (oocytes: *R*^2^ = 0.969, *P* < 0.001; livers: *R*^2^ = 0.850, *P* < 0.001in livers; fig. 2). Within the oocyte data set, the position of individuals almost linearly followed their genome composition; that is, the global transcription profiles of diploid hybrids appeared truly intermediate. By contrast, the patterns observed in liver tissue were similar to those of morphological and habitat preference data where the diploid hybrids were not fully intermediate and all hybrids generally tended to be disproportionately similar to *C. taenia* (fig. 2 and supplementary fig. 2, Supplementary Material online).

In summary, we found that genomic dosage significantly affects the phenotypic similarity of hybrid di- and poly-ploids to their parental species in all data sets, but the effect was much weaker in external phenotype (morphology and habitat preferences) than in gene expression. Also, there were notable differences between germinal traits (oocyte expression profiles) where the appearance of hybrids strictly reflected their genomic dose, and “somatic” traits (morphology, habitats, and liver expression profiles) where the hybrid’s appearance was consistently skewed toward one parental species.

### The Effects of Hybridization and Polyploidy on Expression Regulation of Individual Genes

#### Expression Evaluation of Individual Genes According to Parental Species Expression Levels

**Fig. 4.**
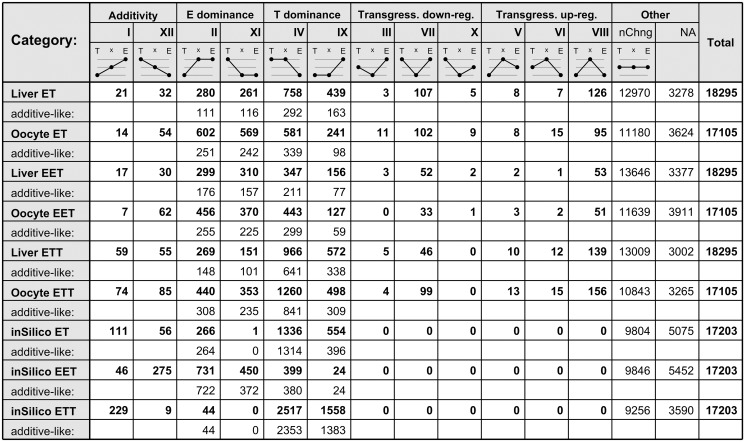
Expression-level categories according to parental expression levels. For each biotype and tissue data set (including the in silico hybrids), we demonstrate the number of genes falling into one of the 12 expression inheritance categories sensu (Yoo et al. 2013). Genes with no significant change among any pair of biotypes were listed as “nChng,” whereas those with ambiguous categorization were listed as “NA.” For the expression-level dominance categories, we listed the number of so-called “additive-like” genes, that is, the cases where hybrids were not significantly different from the dominant parent, but their FC indicated rather intermediate position (note that in real data such cases represented ∼50%, whereas for in silico hybrids they constituted over 95% of such cases). The pictograms in the “table” schematize the respective gene expression pattern for each category: black dot on the left indicates the relative expression of *Cobitis taenia* and is denoted by “T,” the dot on the right indicates *C. elongatoides* (“E”), and the dot in the middle indicates relative expression of given hybrid biotype.

In this section, we examined the expression regulation of individual genes and the effect of such individual gene regulation on the overall intermediacy of hybrids and the apparent genomic dosage gradient. Specifically, we investigated how each gene was expressed relative to its level in parental species. To do so, we classified individual genes in each hybrid biotype into expression inheritance categories sensu Yoo et al. (2013), see figure 4 for visual description of categories, which assume additivity (categories I and XII) if the given gene’s expression significantly differed between both parental species, whereas the hybrid’s expression was intermediate, transgressivity when the hybrid’s expression was significantly inferior (categories III, VII, and X) or superior (categories V, VI and VIII) to both parental species or expression-level dominance (categories II, IV, IX, and XI) when both parental species significantly differed from each other but the hybrid significantly differed from one but not the other parent.

The majority of studied genes (∼12,000 in each biotype) showed conserved expression with no significant variability among any pair of compared biotypes, but ∼6,000 genes showed significant differences between at least one pair of biotypes. About 50% of these genes could not be categorized according to Yoo et al. (2013) as only one of the three possible pairwise comparisons yielded significant differences. Such ambiguous genes were generally characterized by low fold change (FC) differences between the parental species. The remaining ∼3,000 genes were successfully assigned to one of the 12 inheritance classes and in majority, their assignment was consistent among biotypes since there was a highly significant correlation among matrices of category sharing/transition between all pairs of biotypes (Mantel test *P* < 0.01 in all cases). Only a minor fraction of classified genes showed biotype-specific switches between related categories, which occurred between inheritance expression categories characterized by identical FC between parental species but differing in the position of hybrids (e.g., the given gene was assigned to class II in EET but into the class I or IX in ET or ETT hybrids).

As mentioned above, only a small proportion of genes showed transgressive patterns (5.8–12.6%) or additive expression (2.6–5.2%; fig. 4). By far the greatest proportion of genes with assigned inheritance category displayed expression-level dominance (83–89.7%). Two interesting patterns emerged among dominantly expressed genes. First, *taenia*-expression-level dominance was more common in liver tissue than in oocytes of all biotypes, which is consistent with the aforementioned nonlinearity of the genomic dosage effects. Second, more genes in triploids appeared under expression-level dominance of the species which contributed double genomic dose.

We have to consider the possibility of artifactual gene assignments due to data distribution and a lack of statistical power. For example, additively expressed genes might have been misclassified into the dominance class if we lacked significant distinction between a tested hybrid and one of its parents. In such cases, however, the FC between hybrids’ and parentals’ gene expression would still tend to appear additive-like, that is, although hybrids’ expression would not significantly differ from the dominant species, it would still be shifted in the direction of the other parental species. In other words, hybrids would still appear within rather than beyond the ranges of both parental species. This artifactual behavior was evident in simulated hybrids where over 92% of their genes assigned to expression dominance possessed such additive-like patterns (fig. 4). By contrast, in real hybrids, depending on tissue and biotype, only 47–64% of their genes assigned to expression-level dominance had such an additive-like pattern. Moreover, in comparison with simulated hybrids, the proportion of genes assigned to additivity significantly differed (contingency table *P* value <0.01) and was ∼5 times less frequent. This suggests that our gene assignments have not been largely affected by statistical artifacts and observed data indicate a significant lack of additively expressed genes.

In summary, hybrids expressed relatively few genes at levels transgressing those observed in their parents and surprisingly, even less genes were expressed at intermediate levels (additivity). Instead, expression-level dominance vastly prevailed and there were clear differences between tissues (significant prevalence of *C. taenia* dominance in livers) and ploidies (in triploids the prevalent dominance was attributable to the parent contributing two chromosome sets).

#### Relative Allelic Expression and Cis- versus Trans-Regulation

To further explore the molecular causes of transcription modulation induced by hybridization and polyploidy, we analyzed how the parental alleles (homoeologs) are expressed within each gene and whether their relative expression corresponds to the expression divergence between parental species. The expression of genes is presumably under control of interacting cis- and trans-regulatory factors and hence the relative allelic expression (RAE) may be affected by cis-/trans-regulatory divergence between parental species since both alleles in hybrid cells are exposed to the same set of trans-regulatory factors. We therefore tested whether hybrids generally conserved the unbalanced parental expression or deviated from it in some specific way.

To do so, we first selected genes where parental transcripts could be diagnosed due to the presence of species-specific single nucleotide polymorphisms (SNPs) and assigned each allele as *elongatoides*-specific (denoted as Ehyb in following text) or *taenia*-specific (denoted as Thyb). Subsequently, we applied negative binomial generalized linear models (GLMs) to test for regression of Ehyb/Thyb ratio to E/T divergence across all such genes. Note that in the case of triploids, the ratios were appropriately adjusted to take into account a 1:2 or 2:1 genomic dose of alleles.

**Fig. 5.**
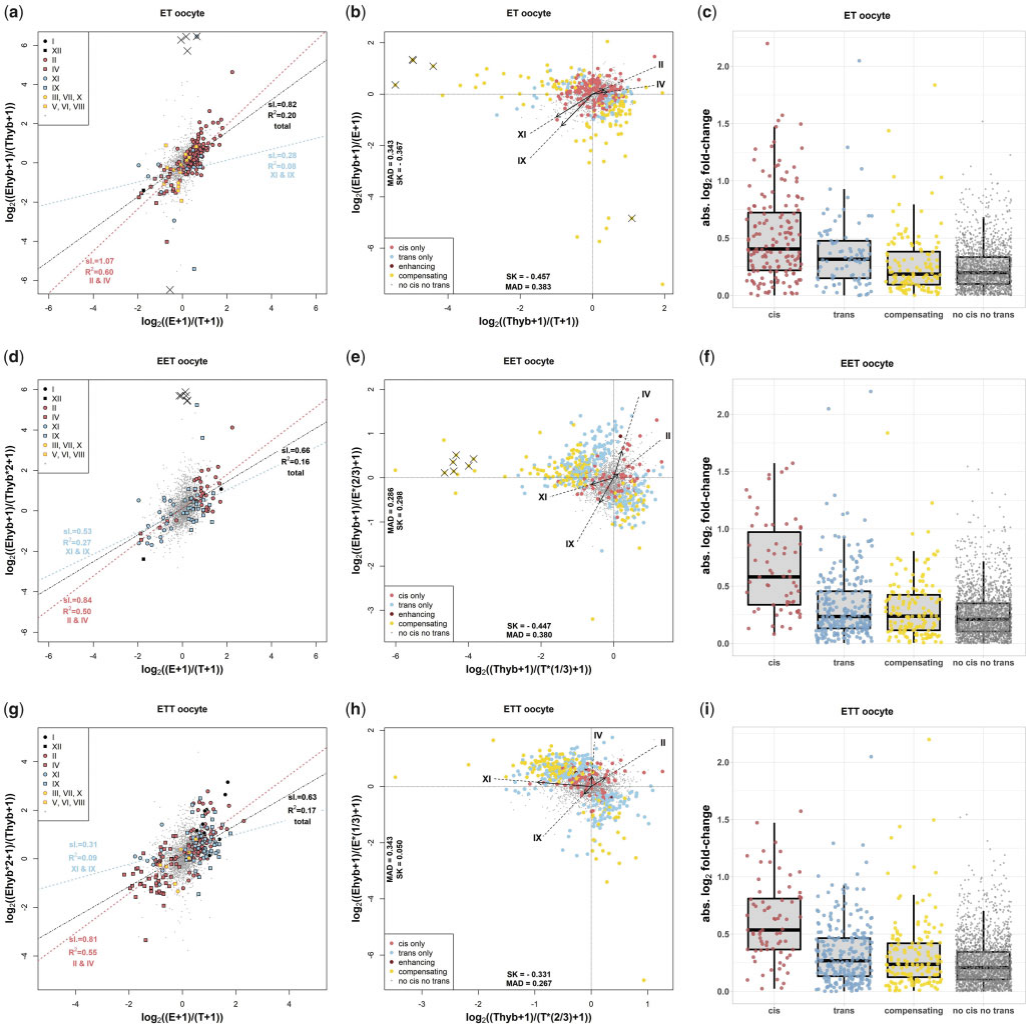
Cis- and trans-regulatory divergence between parental species and hybrids. (*a*, *d*, and *g*) Scatterplots demonstrate the relative ASE levels in parents (E/T on the *x* axis) and hybrids (Ehyb/Thyb on the *y* axis; E, T and Ehyb, Thyb correspond to read counts in parents and hybrids’ allelic, respectively). (*a*) Scatterplots for 2,024 genes in ET hybrids, (*d*) scatterplots for 2,024 genes in EET hybrids, and (*g*) scatterplots for 2,683 genes in ETT hybrids. Each point represents a single gene on a logarithmic scale and is color coded according to the inheritance expression category by Yoo et al. (2013); genes which could not be assigned are shown in gray and genes with complete homoeolog silencing are indicated by cross sign. Three regression slopes are demonstrated with corresponding statistics for the total data set, for genes with expression-level dominance UP and expression-level dominance DOWN patterns, respectively (see Materials and Methods). (*b*, *e*, and *h*) Scatterplots demonstrate variation in ASE where each point represents a position of a single gene with respect to the combined up- and down-regulation of both alleles (Ehyb/E on the *x* axis; Thyb/T on the *y* axis; Ehyb, Thyb and E, T correspond to read counts of hybrid’s alleles and parentals, respectively). (*b*) Scatterplots for 2,024 genes in ET hybrids, (*e*) scatterplots for 2,024 genes in EET hybrids, and (*h*) scatterplots for 2,683 genes in ETT hybrids. The colors show the attribution of each gene to cis-only, trans-only, enhancing and compensatory category with gene not attributed shown in gray. The four arrows indicate the position of a centroid for genes belonging to four expression-level dominance categories sensu (Yoo et al. 2013). Note that coordinates of centroids usually indicate greater deviations from 0 along the axis defined by the nondominant homoeolog. (*c*, *f*, and *i*) The box plots demonstrate the magnitude of absolute (FC) parental expression divergence attributable to cis, trans, and cis + trans compensating interactions. (*c*) Results in ET hybrids, (*f*) results in EET hybrids, and (*i*) results in ETT hybrids. In all hybrid biotypes, there was significantly higher expression divergence in cis-regulated genes than in other types of regulation (Wilcoxon’s rank-sum test, *P* < 0.001) and in ET biotypes we also found significant difference between trans and cis + trans compensating categories (Wilcoxon’s rank-sum test, *P* < 0.001).

Altogether, depending on expression levels ensuring sufficient coverage for SNP calling, we investigated between 873 and 2,681 genes per data set and found that complete silencing of one parental allele was very rare. Instead, both parental alleles were expressed in more than 99% of genes in all data sets. The overall distributions of normalized RAE values were significantly correlated with the expression divergence between parental species (fig. 5*a*, *d*, and *g* for oocytes and supplementary fig. 3*a*, *d*, and *g*, Supplementary Material online, for livers; GLMs *P* value <10^−5^ in all data sets). This either indicates that cis-regulation is pervasive in all data sets, or that cross-talk between trans-elements from both genomes has been hampered and the expression of homoeologs mostly obeys their own genome-specific signals. Nonetheless, the percentage of RAE variation explained by interspecific expression divergence was rather low and corresponding *R*^2^ values ranged from 0.07 to 0.2, depending on the data set. By contrast, such correlation was much stronger in the in silico simulated hybrids (*R*^2^ of linear models applied to in silico hybrid data varied from 0.5 to 0.65). Such a difference between real and simulated values suggests that a nonnegligible proportion of genes deviated from expectations under pure cis-regulation, with a tendency to either equalize the allelic expression (trans-regulation patterns) or magnify the differences between homoeologs (cis-/trans-compensation).

To refine such an observation, we post hoc categorized individual genes in all hybrid biotypes into cis-/trans-categories sensu (Shi et al. 2012). These categories assume *undifferentiated expression* when allelic expression between parents and within hybrids is similar and balanced (i.e., E = T and Ehyb/Thyb = 1), *cis-regulation* when allelic expression between parents is unbalanced and preserved within hybrids (E/T <> 1 and Ehyb/Thyb = E/T), *trans-regulation* when allelic expression in hybrids is balanced despite differentiation between parents (E <> T and Ehyb/Thyb = 1), or, lastly, with *combined cis + trans effects* depending on the direction of cis- and trans-effects. Most of the genes in which differentiation between parentals offered sufficient power to reject both null hypotheses (between 247 and 488 genes depending on data set) were attributed to either cis-only, trans-only or cis + trans compensating categories. The cis + trans enhancing category was rare in all biotypes. We found no enriched GO terms in either cis- or trans-only regulated groups, which prevents speculation of the role of cis-/trans-regulation in particular genetic pathways.

We show in following four paragraphs that relative efficiency of cis- and trans-regulation followed several notable trends:

First, the *proportion of cis- and trans-regulated genes was significantly affected by ploidy*. This was apparent from the fact that the proportion of cis-only genes was similar in both EET and ETT triploids, but it was significantly higher in diploid hybrids (table 1, contingency table test *P* < 0.001 in all comparisons). We also observed higher *R*^2^ of the Ehyb/Thyb ∼ E/T regression model in ET diploids than in both triploid forms again suggesting stronger cis-regulation in diploids (fig. 5*a*, *d*, and *g* for oocytes and supplementary fig. 3*a*, *d*, and *g*, Supplementary Material online, for livers).

**Table 1.** Expression Regulatory Categories: Frequency of Genes in Each Regulatory Divergence Category for Hybrid Biotypes.

Biotype/tissue	Cis	Trans	Enhancing	Compensating	NoCisNoTrans	NotAvailable
**ET (oocyte)**	150	86	0	124	1,664	15,081
**EET (oocyte)**	62	244	2	147	2,226	14,424
**ETT (oocyte)**	74	251	3	160	2,195	14,422
**ET (liver)**	70	94	4	79	626	17,422
**EET (liver)**	34	233	2	96	1,099	16,831
**ETT (liver)**	34	237	1	118	1,127	16,778

Note.—For each biotype and tissue data set, we demonstrate the number of genes falling into one of the expression-regulation categories. Genes where no diagnostic SNP was present or which did not met criteria for *cis*-/*trans*-categorization were listed as “NotAvailable”.

Second, the *efficiency of cis-regulation was tissue-specific* as evidenced by the fact that all biotypes displayed significantly a higher proportion of cis-only regulated genes in oocytes than liver tissues (table 1, contingency table test *P* < 0.005 in all comparisons). Additionally, the *R*^2^ of the Ehyb/Thyb ∼ E/T regression model was higher in oocytes than in livers of all biotypes (fig. 5*a*, *d*, and *g* for oocytes and supplementary fig. 3*a*, *d*, and *g*, Supplementary Material online, for livers).

Third, we discovered an *effect of type of expression inheritance*. In particular, when focusing on dominantly expressed genes (categories II, IV, IX, and XI), we found that RAE in hybrids (Ehyb/Thyb) is not only significantly correlated to parental expression divergence (E/T) but there was also significant interaction with the attribution of the given gene to inheritance categories assuming expression-level dominance UP versus expression-level dominance DOWN (categories II + IV vs. IX + XI; the simpler GLM with formula Ehyb/Thyb ∼ E/T was significantly outperformed by a GLM with formula = Ehyb/Thyb ∼ E/T + E/T*inheritance_category). In other words, parental expression divergence is more conserved in expression-level dominance UP category than in expression-level dominance DOWN. Figure 4*a*, *d*, and *g* demonstrates this effect as the regression slopes were significantly steeper (*∼1*) and related *R*^2^ significantly higher in categories II and IV than in categories IX and XI, where the RAE was more balanced and hence the trans-effects appeared relatively stronger. Nonetheless, the aforementioned interaction was significant in oocytes of all biotypes, but not significant in livers.

Finally, *cis- and trans-regulation differed between genes according to the parental expression divergence*. Specifically, cis-only regulation was more likely to occur in genes with higher parental expression divergence than other categories involving trans-effects (fig. 5*c*, *f*, and *i* for oocytes and supplementary fig. 3*c*, *f*, and *i*, Supplementary Material online, for livers).

#### Up- and Down-Regulation of Homoeologs

Finally, we compared the absolute expression of each species-specific allele in hybrids to its expression value in given parental species (i.e., we investigated the Ehyb/E and Thyb/T ratios), which is a useful tool to investigate magnitudes of homoeolog expression modulation. Several important patterns emerged from this investigation.

First, *homoeolog downregulation prevailed over upregulation*. Specifically, although the log_2_-distributions of Ehyb/E and Thyb/T were centered at zero in all biotypes, suggesting generally conserved expression of most homoeologs (fig. 5*b*, *e*, and *h* for oocytes and supplementary fig. 3*b*, *e*, and *h*, Supplementary Material online, for livers), they were significantly negatively skewed (only in oocyte data of EET and ETT the negative skewness of log_2_[Ehyb/E] was not significant). This suggests that whenever homoeolog expression modulation occurs, the downregulation causes higher FC between hybrids and parentals than upregulation.

Second, the magnitude of homoeolog expression modulation was *tissue specific*. Namely, we noticed that the amount and direction of trans-effects between both genomes were significantly correlated between tissues in all biotypes (all *P* values of correlations between log_2_ of Ehyb/E and Thyb/T ratios between oocyte and liver data were lower than ≪10^−6^, whereas *R*^2^ ranged from 3% to 33%). However, overall dispersion of allelic expressions in hybrids relative to parental species was significantly higher in livers (Fligner–Killeen test *P* < 10^−13^ in all biotypes). In other words, the absolute allelic expressions were centered at zero in both tissues and all biotypes, but when a particular homoeolog of a given gene was modulated in one tissue, the other tissue usually showed the same trend. However, absolute amounts of deviations from parental values were higher in liver tissue, suggesting that hybrids generally modulated the allelic expressions in liver tissue to a greater extent than in oocytes.

Third, the *efficiency of trans-expression modulation depended on the genomic dosage*. In diploids, the effects of E trans-regulatory factors on T homoeolog expression and vice versa were apparently more symmetrical than in triploid hybrids, where the effects of one parent’s trans-factors were always prevailing over the other’s (fig. 5*b*, *e*, and *h* for oocytes and supplementary fig. 3*b*, *e*, and *h*, Supplementary Material online, for livers). Specifically, in diploids the log_2_(Thyb/T) ratios were significantly more dispersed from zero than log_2_(Ehyb/E) (Fligner–Killeen test of median absolute deviation [MAD]; *P* < 10^−3^ in both tissues). This suggests that E trans-regulatory factors have a greater effect than T trans-regulatory factors on RAE variation. However, the distributions of both log_2_(Ehyb/E) and log_2_(Thyb/T) ratios were negatively skewed to a similar extent (permutation test on skewness, *P* values >0.1 in both liver and oocyte tissues), suggesting the differences were rather small. The situation was strikingly different in triploids. In triploids we found highly significant differences between log_2_(Ehyb/E) and log_2_(Thyb/T) ratios both in terms of skewness (permutation test on skewness, *P* values <0.001 in all triploid data sets except ETT in livers where *P* value =0.18) and MAD (Fligner–Killeen test *P* ≪ 10^−8^ in all triploid data sets). In particular, both EET and ETT biotypes had significantly greater deviations from parental expression levels in those homoeologs that originated from the parental species contributing a haploid set of chromosomes. This suggests that homoeolog expression regulation of the “haploid” parental genome is more pronounced than that of the “diploid” genome, regardless of whether it is *C. taenia* or *C. elongatoides*.

To further explore the dosage dependency of the trans-effects, we compared the dispersion of log_2_(Ehyb/E) and log_2_(Thyb/T) between diploid and triploid hybrids. There were no significant differences between diploids and triploids in the log_2_ expression ratios of those alleles, which are haploid in triploids (e.g., log_2_(Ehyb/E) did not differ between ET and ETT biotypes). By contrast, there were significant differences in the log_2_ expression ratios of those alleles, which are duplicated in triploids (Fligner–Killeen test *P* ≪ 10^−11^), for example, log_2_(Ehyb/E) differ between ET and EET biotypes. This suggests that “haploid” homoeologs in triploids are modulated by trans-regulatory factors to a similar extent as those in diploid hybrid biotypes. However, homoeologs occurring in triploid genomes with double dosage are less affected by trans factors than if occurring in diploid hybrids.

## Discussion

### Hybrids and Polyploids Lack Transgressivity. Their Overall Intermediacy Results from Nonadditive Expression

Genome merging may affect hybrids’ phenotypes both positively and negatively, for example, by inducing evolutionary novelties, “genomic shock” or karyotype instability (Bell and Travis 2005; Dion-Côté et al. 2014; Ficetola and Stöck 2016; Lien et al. 2016), and the severity of its effects probably correlates with the divergence between parental species. Yet, in spite of substantial expression differentiation that evolved during more than 9 Mya since speciation between *Cobitis* parental species (Janko et al. 2018), merging of their genomes provoked only little phenotypic transgressivity in hybrids and <10% of their genes were categorized as transgressive according to expression inheritance categories. Similarly, polyploidy clearly impacts some important traits in *Cobitis*, such as fecundity or metabolism (Juchno and Boron 2010; Maciak et al. 2011), however, it did not induce any pervasive transgressivity of gene expression. It is worth noting that no consistent differences between di- and poly-ploids have been identified by other studies of fish, even when using the same tissues we did. Specifically, whereas Li et al. (2014) and Luo et al. (2015) reported prevailing upregulation of DEGs in polyploid *Misgurnus* and *Carassius*, the opposite patterns of prevalent downregulation were reported by Matos et al. (2015) and Ren et al. (2017) in polyploid *Squalius* and *Ctenopharyngodon idellus*–*Megalobrama amblycephala* hybrids.

We could not compare parental specimens and their direct F1 progeny, which are difficult to obtain (Choleva et al. 2012). Thus, observed patterns may reflect not only the immediate effects of hybridization or polyploidy but also the effects of postformational changes, such as selection against transgressive phenotypes, plastic effects of environment, or particular combinations of alleles in studied hybrids. However, this should not alter our conclusion that hybridization and polyploidy per se did not provoke pervasively novel trait expression since examined clones are of recent origin (Janko et al. 2012) and accumulating postformational changes are expected to shift hybrid biotypes to new forms, henceforth increasing the transgressivity rather than decreasing it (Yoo et al. 2014). Instead, the morphological, ecological and gene expression variability of hybrids mostly oscillated within the limits determined by their parental species and was significantly affected by the dosage of parental genomes.

Rather surprisingly, the overall intermediacy of *Cobitis* hybrids did not stem from pervasively additive gene expression as >83% of genes with assigned inheritance category indicated expression-level dominance. Such patterns are especially striking in comparison to other organisms, including fish, that reported considerably higher proportions of additively and transgressively expressed genes (Bell et al. 2013; Yoo et al. 2013, 2014; Combes et al. 2015; Matos et al. 2015; Meyer et al. 2012; Ren et al. 2017; Schedina et al. 2018).

To some extent, the focus on particular tissues might have affected our conclusions but gene expression in livers is frequently investigated (Goncalves et al. 2012; Gao et al. 2015; Wong et al. 2017; Zhuo et al. 2017; for fishes see Li et al. 2014; Matos et al. 2015; Ren et al. 2017) and similarly, the analysis of ovarian tissue is a logical step given the asexual reproduction of *Cobitis* hybrids and may be compared with other studies investigating this kind of reproduction (Li et al. 2014; Luo et al. 2015; Schedina et al. 2018). Additionally, an occasional lack of statistical power might have induced misclassifications of additively expressed genes into the dominant class. However, differences between empirical and simulated data corroborate the general lack of additive expression in *Cobitis*, suggesting that observed patterns are most likely not artifactual.

The mechanisms underlying hybrid phenotypes have only recently started to be uncovered, but accumulating evidence indicates that many genes are expressed nonadditively (Yoo et al. 2014). Naturally, most research has been directed toward cases where hybridization has induced the expression of novel traits. Yet, the present study documented that the *intermediate appearance of hybrids is coupled with extraordinarily pervasive expression-level dominance*. Thus, our data imply that intermediate phenotypes do not necessarily result from a simple summation of products of individual genomes, but rather from complex interactions of regulatory networks and combinations of individual genes, which are expressed at levels typical for either one parent or the other parent, but not at an average level.

### The Role of Cis- and Trans-Effects in Gene Expression Modulation of Hybrids and Polyploids

How hybrids transcript their genes ultimately depends on the transcription of individual homoeologs, which are governed by interacting cis- and trans-regulatory factors from both parental genomes. Strictly speaking, the effects of cis-regulatory elements are expected to be limited to within one parental genome, whereas trans-regulatory elements affect both parental genomes. Categorization of genes as cis- or trans-regulated is usually based on comparison of RAE to the expression divergence between parents (Shi et al. 2012). However, unless one is able to limit the analysis to relatively short genomic fragments of one species inserted onto an allospecific genomic background (e.g., Meiklejohn et al. 2014), one should be aware, that such categorization is somewhat ambiguous in hybrids. This is because genes regulated by trans-acting factors might respond to their species-specific factors and therefore appear as cis-regulated when the cross-talk between merged genomes is hampered. Such a situation nonetheless points at regulatory divergence between parental species.

Our data indicated pervasive cis-regulation (or divergence as mentioned above) in all hybrid/polyploid data sets (fig. 5). However, the correlation of RAE with interparental expression divergence was much weaker than expected under pure cis-effects since the regression *R*^2^ was considerably higher in simulated data (0.5–0.65) than in real hybrids (0.07–0.2). Such a noisy distribution around fitted values implies frequent modulation of *elongatoides* alleles by *taenia* trans-regulatory elements and vice versa. The negative skewness of Ehyb/E and Thyb/T ratios further suggests that downregulation has more pronounced effects than upregulation. Nevertheless, complete allele silencing seems rare as both homoeologs were expressed in more than 99% of genes (fig. 5 and supplementary fig. 3, Supplementary Material online).

Our data generally agree with the premise of Yoo et al. (2013) and Combes et al. (2015) that expression-level dominance is triggered by trans-regulation which induces greater expression modulation of alleles derived from the nondominant parent than of those derived from the dominant one. This is apparent from generally greater deviations from the parental expression levels in the nondominant homoeologs as indicated by the positions of centroids in Ehyb/E ∼ Thyb/T distributions for the expression-level dominance categories II, IV, IX, and XI (fig. 5*b*, *e*, and *h* for oocytes and supplementary fig. 3*b*, *e*, and *h*, Supplementary Material online, for livers).

However, the reality is more complex as the efficiency of trans-regulation also depends on the amount of divergence between the parental species (rev. in Yoo et al. [2014]) and even varies among individual genes within the single hybrid strain depending on the interparental divergence in those particular genes. Specifically, the results of Shi et al. (2012), Meiklejohn et al. (2014), and Combes et al. (2015) as well as our own data all agree on the fact that groups of genes assigned to pure-cis, pure-trans or cis + trans combined regulation significantly differ in FC between parental species. Quite surprisingly, however, these studies disagree on the direction of the effect of interparental FC since trans-regulated genes in *Cobitis* (fig. 5*c*, *f*, and *i*;supplementary fig. 3*c*, *f*, and *i*, Supplementary Material online) and *Drosophila* (Meiklejohn et al. 2014) have generally lower interparental FC than cis-regulated ones, whereas *Coffea* plant hybrids showed the opposite trend (Combes et al. 2015) and Shi et al. (2012) reported no differences between cis-only and trans-only regulated *Arabidopsis* genes, but lower divergences in the cis + trans compensating category. Such controversy over the effect of interparental expression divergence on the cis-/trans-regulation demonstrates a current gap in the understanding of underlying mechanisms.

Furthermore, we found that the relative contributions of cis-/trans-regulatory mechanisms systematically differed among expression inheritance categories. In particular, the genes with expression-level UP dominance (categories II and IV) were under stronger cis-regulation than the genes with expression-level DOWN dominance (categories IX and XI), whose RAE was significantly more equilibrated (fig. 5*a*, *d*, and *g*). To our knowledge, such differences have not been explicitly mentioned to date, but similar patterns stem from published data of *Coffea* plant hybrids (Combes et al. 2015) suggesting that our observation may have a general validity. The expression regulation of homoeologs thus appears to be different in genes where the dominant genome is upregulated from those where it is downregulated.

### Polyploidy Affects the Genomic Dosage and Modifies Cis-/Trans-Expression Regulation

Although polyploidization per se did not induce much transgressivity, it considerably affected *Cobitis* hybrids by changing their genomic dosage, since triploids generally appear more similar to the parental species contributing the double genomic dose. Similar patterns were also reported from other allopolyploids (e.g., Chen and Ni 2006; Kierzkowski et al. 2011; López-Caamal and Tovar-Sánchez 2014), but the underlying mechanisms remain elusive. We propose a new potential explanation for this phenomenon, since our data indicated the appearance of hybrids and polyploids did not result simply from additive expression of parental alleles. Instead, polyploidization appears to modify the efficiency of cis-/trans-regulation. Specifically, we observed a higher proportion of cis-only regulated genes in diploids, whereas more genes in triploids were categorized as trans-only regulated (table 1). Also, RAE in diploids matched parental expression divergence more strongly than in triploids (fig. 4*a*, *d*, and *g*). Altogether, this implies stronger cis-regulation in diploids than in triploids, where trans-regulation of homoeolog expression was more efficient.

Explanations for this observation are unclear but relate to the fact that expression modulation of homoeologs depended on their genomic dose. In particular, diploids possessed almost symmetrical distributions of log_2_(Ehyb/E) and log_2_(Thyb/T) ratios, whereas in triploids these distributions were significantly asymmetrical and homoeologs originating from the diploid genome were less modulated by the other genome’s trans-regulatory factors than vice versa. Importantly, such effects were observed in both EET and ETT triploids suggesting that asymmetry in trans-effects does not depend on a particular combination of parental genomes but rather on their dosage. To our knowledge, such effects of polyploidy have not been published, but Veitia et al. (2013) suggested that the efficiency of trans-regulators is proportional to their concentration relative to the binding sites. If so, then our study indicates that in triploids, the trans-regulators derived from the parental species contributing two genomes exert more effects on the respective gene regions from the other parent than vice versa. Phenotypic consequences of genomic dosage in polyploids may thus partly stem from modified relative concentrations of trans-regulators and the respective genetic regions of both hybridizing genomes.

Interestingly, the observed genomic dose gradient was nonlinear with hybrids being disproportionately similar to *C. taenia* in morphology, habitat selection and gene expression in liver tissue. Such expression bias is known from other allopolyploids (e.g., Grover et al. 2012; Alexander-Webber et al. 2016) and proposed explanations include stronger expression regulation of one parental genome due to unequal distribution of transposable elements, or different specificity of homoeologous trans effectors to target genes (Bottani et al. 2018). The expression bias may affect the subsequent genome evolution of neopolyploids, since the nondominant genome may be more prone to elimination during the rediploidization process (Schnable and Freeling 2012). Although this process may influence the future genome evolution of *Cobitis* hybrids, it is clear that current *C. taenia*-biased expression-level dominance does not result from silencing or the loss of *elongatoides* alleles since both homoeologs were expressed in more than 99% of scored genes. The bias may also result from nuclear-cytoplasmic interactions (Wolf 2009; Yoo et al. 2014). Unfortunately, predominantly unidirectional hybridization in *Cobitis* prevents us from testing such a hypothesis since hybrids with *elongatoides* mitochondrion are rare (Janko et al. 2012).

In any case, our study adds two important aspects to the phenomenon of biased expression-level dominance. First, the bias was present in all biotypes in the morphological, ecological, and liver-expression data sets suggesting it does not depend on a particular ploidy level. Second, the genomic dose gradient in oocytes was nearly linear with no evident *taenia* dominance, suggesting that interactions between hybridizing genomes are tissue specific.

### Concluding Remarks and Outlook

Hybridization and polyploidy have been intensively investigated particularly for their potential to generate evolutionary novelties, but even “normal” nontransgressive hybrids may establish successful lineages with considerable evolutionary potential. Hybrid “intermediacy” may be important especially for asexual organisms, whose successful establishment is facilitated by inheriting a narrow segment of parental variability without a need to adapt for different niches via evolving new traits (e.g., Vrijenhoek 1979; Janko et al. 2011; Mau et al. 2015). Additional advantage in competition with sexual counterparts and diploid clones may come from polyploidization (Beukeboom and Vrijenhoek 1998; Janko et al. 2012) which systematically affects the dosage of genes and, as we show, even the type of expression regulation of alleles.

The success of di- and poly-ploid hybrids depends on, among other things, the genetic divergence between their parental species (e.g., Russell 2003; Bolnick and Near 2005; Chapman and Burke 2007; Paun et al. 2009). Tulchinsky et al. (2014) suggested that this effect may relate to the divergence in cis-/trans-regulatory elements. The type of hybrid’s reproduction (such as asexuality) also appears to depend on interparental divergence (see Janko et al. 2018 and references therein) and our data therefore indicate several promising research directions.

For example, Carman’s (1997) widely cited hypothesis assumes that alterations of gametogenesis toward clonality are caused by asynchrony of diverged developmental programs that are autonomously executed in a hybrid. In our opinion, such autonomous execution is possible only when cross-talk between genomes is hampered, otherwise homoeolog expression would be synchronized by trans-regulation. Given that efficiency of trans-regulatory cross-talk negatively correlates with genetic divergence, this may also explain why distant rather than close species must breed to produce asexual hybrids. Furthermore, our observation that the efficiency of trans-regulators depends on their genome dosage may help to explain why the stability of asexual lineages is so dependent on ploidy (see e.g., Moritz et al. 1989; Choleva and Janko 2013): particular ploidy levels may determine the extent to which both genomes cross-regulate each other’s transcription or maintain their autonomy—a necessary assumption of the Carman’s (1997) model.

In addition, the evidence of different cis-/trans-regulation patterns between oocytes and livers may have important implications, especially in relation to the recent discovery of tissue-specific activity of transcription factors (GTEx Consortium et al. 2017; see Bottani et al. 2018 for potential explanation of such a phenomenon). Potentially hybridizing genomes can maintain higher levels of autonomy in the germline through stronger cis-regulation, thereby maintaining proper asynchrony of gametogenetic programs leading to clonality sensu (Carman 1997), whereas more efficient trans-regulation in soma may enable tissue-specific choice of expression-level dominance thereby enhancing organismal plasticity (Buggs et al. 2014).

We propose the understanding of the general crosslink between hybridization, asexuality, and polyploidy may be considerably boosted by explicit consideration of the interplay between cis- and trans-regulation.

## Materials and Methods

### Collection of Fish Material

Fish were captured by standardized electrofishing across the Odra R. hybrid zone, at sites, where previous analysis by Janko et al. (2012) identified the presence of required biotypes. We excised small piece of fin tissue from all fish and performed routine taxonomical identification using species-diagnostic PCR-RFLP and allozyme markers (Janko, Flajšhans, et al. 2007). The ploidy was determined by erythrocyte measurements based on blood sampled from the fin clip (Kotusz 2008). Subsequently, fish were either released back, anesthetized, and preserved in 4% formalin for morphological examination or transported to laboratory for subsequent analysis of gene expression. Fish for gene expression analyses were sampled at beginning of reproductive season (late May) to ensure the presence of oocytes at the developmental stage VI, but to minimize the impact of actual fish state and local conditions, they were maintained for several weeks at standardized conditions at our aquarium set (water recirculation at ∼20 °C, tanks of size 30 × 40 × 25 cm with five individuals per tank) and fed by tubifex before sacrification.

### Collection of Morphological Data

Morphological analysis of complete body shapes involved 191 females (38 belonged to TT, 24 EE, 95 diploid ET hybrids, and 34 triploids—17 EET and 17 ETT individuals). Males were excluded from analysis due to sexual dimorphism in body shape (Kotusz 2008) and because they are sterile in hybrid state, thereby not contributing to the maintenance of asexuality (Janko et al. 2012, 2018). Specimens were photographed from the left side and TpsDig v.1.39 (Rohlf 2003) was subsequently used to digitize 21 homologous landmarks (fig. 1*b*). Graphical representation of average shape of parental species (TT, EE) was based on generalized Procrustes analysis (Rohlf and Slice 1990) in MorphoJ v 1.05 software (Klingenberg 2011). Generalized Procrustes analysis for subsequent statistical analysis was performed in R (R Core Team 2018) using geomorph package (Adams et al. 2018).

### Collection of Microhabitats Data

To assess the association of each individual with its habitat characteristics, we used the method of Point Abundance Sampling (e.g., Pekárik et al. 2012), which allowed their precise localization based on *Cobitis* typical behavior to burry in the sediment. At each sampling point with *Cobitis* sp. presence, we recorded following microhabitat parameters according to (Pekárik et al. 2012): water depth measured to nearest cm, average water velocity measured with SchiltknechtMiniAir 20 velocity meter, relative distance from bank (i.e., the ratio of the distance of given point from the nearest bank and the width of the stream), illumination (evaluated as directly illuminated by the sun or shaded by the debris), proportion of different substrata types at given site, and the type of available refuges (e.g., stones and plants). To minimize the effect of large values in our analyses, all variables were rescaled to the values between 0 and 1 (e.g., water depth was recalculated to meters, etc.). Altogether, we sampled 10, 12, 70, 42, and 67 fish belonging to following biotypes: EE, TT, ET, EET, and ETT, respectively.

### Multivariate Statistical Analyses and Evaluation of Genomic Dose Gradients

PCA was used to examine general shape differences among all five biotypes. Discrimination among biotypes was based on CVA of morphological and microhabitat data matrix with Jackknife cross-validation. Significance of differences among biotypes was tested with PERMANOVA followed by pairwise comparison of each biotype. Euclidean distance was used for morphological and RNAseq data, whereas Bray–Curtis distance appeared more appropriate for microhabitat data that were expressed as the ratios. PCA and CVA were performed in R using Morpho package (Schlager 2017).

To evaluate the effect of the genomic dose on the expression of phenotypic traits and genes, we proceeded as follows. We first reduced the *n*-dimensional space by the PCA with Euclidean distance (morphological and gene expression data), or by the PCoA with Bray–Curtis distance (microhabitat data) and evaluated the minimum number of axes significantly explaining the variability within each data set using the Broken-stick model. Subsequently, each individual was characterized by the proportion of E-genome (pure *C. elongatoides* [EE] = 100%, EET = 66.6%, ET = 50%, and ETT = 33.3% and pure *C. taenia* [TT] = 0%) and this so-called E-genomic ratio was than correlated to various combinations of significant ordination axes in each data set in order to find axes that provide the strongest correspondence to the fitted gradient. The significance of correlation was tested using 9,999 permutations, and the result was projected to the relevant ordination biplots.

Furthermore, we were interested in the predicted position of each biotype along the putative genomic dosage gradient. Thus, we have rotated the ordination biplot along the genomic dosage gradient by computing the rotation angle based on the predicted coordinates of the gradient position and this angle was used to rotate the axis 1 and axis 2 coordinates. Afterward, we used Permutational ANOVA to assess whether there are significant differences among biotypes in their positions along the first rotated axis.

### Collection and Evaluation of RNAseq Data

#### RNA Sequencing and Preparation of In Silico Hybrids

We excised liver tissue and gonads of each female immediately after sacrificing. From each gonad, we selected individual oocytes at the same developmental stage VI (i.e., full grown immature oocytes filled with yolk [>1, 1 mm] with the germinal vesicle situated in the oocyte center) and pooled ten oocytes per female to increase the RNA yield. Both tissue types were then placed into TRIzol and frozen. Subsequently, the total RNA was isolated using TRIzol RNA Purification Kit (Thermofisher), and its integrity was determined on Agilent 2100 Bioanalyzer instrument. Only samples with RQI > 8 were further processed. One microgram total RNA was then fragmented and libraries prepared with TruSeq kit (Illumina) with first strand cDNA synthesis conducted from polyA. Sequencing with Illumina HighSeq 2000 – 50-bp SE reads was employed to liver tissue and pools of ten oocytes of 4 EE, 4 TT, 2 ET, 2 EET, and 4 ETT females. To obtain a test data set that could serve as a useful null model for testing deviations from expected expression patterns, we simulated artificial diploid and triploid hybrids in the same number as in natural hybrids. We did so by randomly choosing one TT and one EE parent for each simulated hybrid and then randomly mixed 20 million reads from each parent to obtain simulated diploid hybrid (for simulated triploids we selected the reads from respective parents in 2:1 or 1:2 ratios). These in silico hybrids were than analyzed by identical pipeline as original RNAseq samples.

#### Reference Transcriptome, Read Mapping, and SNP Calling

To map the reads, we used the reference *C. taenia* transcriptome which was assembled and cleaned from potential paralogs by Janko et al. (2018), available at: DDBJ/EMBL/GenBank GGJF00000000.1. For the purposes of present study, the reference was modified in order to incorporate also transcripts potentially missing in *C. taenia* but present in hybrids and *C. elongatoides*. First, we pooled de novo assembled reads from all samples from the present study assembled by Trinity software, 2.0.2 (Grabherr et al. 2011) using the Inchwom method and clustered contigs longer than 300 bp by Markov model (Enright et al. 2002) with granularity parameter = 1.2. The longest contigs from each cluster were than blasted against original reference of (Janko et al. 2018), which provided us with 3,250 new contigs that did not match published transcriptome. The final transcriptome thus contained 18342 contigs (N50 = 1,215, N90 = 612) and BlastN 2.2.31+ verified that 15,355 assembled contigs have counterparts in *Danio rerio* cDNA (Danio_rerio.GRCz10.cdna.all.fa.gz, release 22.7.2017, BlastN hit with bit-score >=80). Annotation of final reference transcriptome is described in the supplementary Methods, Supplementary Material online.

To overcome potential bias stemming from the fact that hybrids’ data with high heterozygosity are mapped onto reference sequence that contains species-specific SNP variants, we masked with *N* all interspecific SNPs previously detected by Janko et al. (2018) and mapped the reads on such masked reference with Mosaik ver. 2014-03-26 (Lee et al. 2014). Reads with mapping quality >=20 were counted by bedtools multicov, v2.26.0 (Quinlan and Hall 2010) and SNPs called by samtools (v. 1.2)/bcftools (call, v. 1.2-151-g7357020)/vcfutils (VarFilter v. 0.9) (Danecek et al. 2011; Li 2011). All SNPs with mapping quality <20, depth <10 and those occurring 3 bp near to indel position were omitted.

#### Differentially Expressed Genes

DEGs were searched only among transcripts with minimal cumulated read counts above 100 to minimize the effect of background expression noise. The data were normalized with the DESeq2 package of R version 3.0 (Anders and Huber 2010) using “per condition” and “maximum” as arguments and DEGs identified by GLMs with negative binomial distribution and *P* values corrected by False Discovery Rate (FDR) method at 0.05 level (Benjamini and Hochberg 1995). Specifically, we tested the effect of three factors: 1) two parental species were contrasted against each other (i.e., EE vs. TT biotypes) to reveal DEGs between the species, 2) sexual versus asexual individuals (i.e., {EE, TT} vs. {ET, EET, ETT} biotypes), and 3) diploid versus polyploid individuals (i.e., {EE, TT, ET} vs. {EET, ETT} biotypes). Since the three factors may interact (note e.g., the large overlap between ploidy and reproduction type when all triploids are asexuals but not all diploids are sexuals), we further employed ANOVA type II using the principle of marginality from R packages MASS (Venables and Ripley 2003) and car (Fox and Weisberg 2010). Here, we investigated the significance of following three factors and their interactions for each gene to tackle: 1) the potential effect of hybrid intermediacy, so that each individual was assigned as either one (EE) or the other (TT) parental species, or as general hybrid {ET, EET, ETT}, 2) the effect of ploidy, so that each individual was categorized either as diploid {EE, TT, ET} or as triploid {EET, ETT}, and 3) the effect of asexuality, so that each individual was categorized either as sexual {EE, TT} or clonal {ET, EET, ETT} form. Note that the last factor differentiates exclusively between reproductive modes (i.e., it contrasts individuals reproducing sexually and asexually), but the first factor explores the differences between both parental species treated separately as well as between their hybrids. All *P* values were FDR corrected at 0.05 level. The expression of several genes was validated by qPCR (see supplementary Methods, Supplementary Material online).

#### Types of Expression Inheritance

Following (Yoo et al. 2013), we post hoc assigned genes into 12 expression inheritance categories depending on how each gene was expressed in given hybrid biotype (ET, EET, or ETT) relative to both its parents (we stress that categorization relative to parentals was performed separately for each hybrid biotype). When no pairwise comparison yielded significant difference, given gene was classified as “No change.” Genes were classified as ambiguous if they could not be attributed to one of the 12 classes (e.g., when both parental species significantly differed from each other but hybrids did not differ from any of them significantly). To evaluate how much the real data deviate from null expectations of pure additivity, we also used contingency tables to detect differences in each biotype between the real data and in silico hybrids.

#### Analysis of ASE and Assignment of Cis-/Trans-Regulatory Divergence

We first sorted the E- and T-specific reads using the database of species-specific SNPs obtained from the variant calls of *C. elongatoides* and *C. taenia* individuals used in this study as well as in phylogenomic analysis of Janko et al. (2018). All SNPs assumed as diagnostic must have met following criteria: 1) successful SNP call in at least two individuals of each species, 2) all scored conspecifics must share the same allele, which 3) differed from all scored individuals of the other species. We thereby identified 46,563 diagnostic SNPs and applied Samtools mpileup, ver. 1.5 software (Li et al. 2009) to extract number of E- and T-specific reads per gene. To alleviate issues of low coverage, we retained only genes with allele-specific coverage >30 in at least two individuals. The counts were normalized using the total count approach and the mean and variance in coverage per gene and biotype was computed. RAE was calculated as allele-specific read counts (Ehyb or Thyb) among the total read counts (Ehyb + Thyb) and expressed as the percentage of Ehyb allele in the total gene expression of individual. As silenced alleles we assumed those that had zero reads, provided that given gene passed above described conditions.

According to Shi et al. (2012), we used two-sided prop.test with FDR correction in R to test if 1) the ratio of expression of both parental alleles in the hybrids corresponds to the expression differentiation between parents (i.e., whether Ehyb/Thyb = E/T), 2) whether the RAE is balanced (i.e., whether Ehyb/Thyb = 1), or 3) whether RAE deviates from both patterns. Namely, if the cis- and trans-effects were in the opposite direction, the gene was categorized as under compensating cis + trans interaction (i.e., Ehyb/Thyb > 1 and E/T < Ehyb/Thyb or Ehyb/Thyb < 1 and E/T > Ehyb/Thyb). Alternatively, if the cis- and trans-effects acted in the same direction, then enhancing cis + trans interaction was assumed (i.e., Ehyb/Thyb > 1 and E/T > Ehyb/Thyb or Ehyb/Thyb < 1 and E/T < Ehyb/Thyb).

#### Up- and Down-Regulation of Hybrid Alleles

We also evaluated the expression regulation of both alleles in genes where ASE information was available. Specifically, we plotted in the log_2_ scale the variation in the expression of each allele in the hybrids and both parents (*x* axis: Thyb/T; *y* axis: Ehyb/E; see fig. 5*b*, *e*, and *h* and supplementary fig. 3*b*, *e*, and *h*, Supplementary Material online, for oocyte and liver data, respectively). Such a graph informs about changes in allelic expression due to trans-regulatory factors coming from the other genome; given the log_2_ scale, when any gene has a negative value along one of the axes, it suggests its allele has been downregulated by the other’s genome trans-regulatory effects, whereas positive values indicate the upregulation. As above, the ratios were adjusted to consider the disbalance in triploids.

To evaluate the prevailing trend (up- or down-regulation) as well as the magnitude of changes in allelic expression, the distributions along each axis were characterized by skewness and MAD (note that distributions were nonnormal and hence MAD was used instead of standard deviation metrics). Wherever it was necessary to evaluate differences between biotypes and/or tissues in terms of log_2_(Thyb/T) or log_2_(Ehyb/E) distributions, we used two approaches. First, the differences in variances between two distributions were tested by comparing their MAD values by Fligner–Kileen test, which is among the most robust nonparametric tests for homogeneity of variances (Conover et al. 1981). Second, the differences between two distributions in prevailing tendencies to negative/positive deviations were tested by comparing their skewnesses using the permutation test. Initially, we recorded the absolute value of difference between skewnesses of the two compared data sets; then, we joined both compared data sets and created pseudoreplicates of original data by random sampling from the joined data set; finally, we estimated the skewnesses of both pseudoreplicates and recorded their absolute difference. Such randomization process was repeated thousand times and the proportion of simulated absolute differences higher than the original value was used as the test *P* value.

## Supplementary Material

Supplementary data are available at *Molecular Biology and Evolution* online.

## Supplementary Material

msz114_Supplementary_Data
