# Supplementary figures and images for: The Legacy of Sexual Ancestors in Phenotypic Variability, Gene Expression, and Homoeolog Regulation of Asexual Hybrids and Polyploids
Source: Mol Biol Evol. 2019 May 11;36(9):1902–20. doi: 10.1093/molbev/msz114 (PMC6735777; doi:10.1093/molbev/msz114)

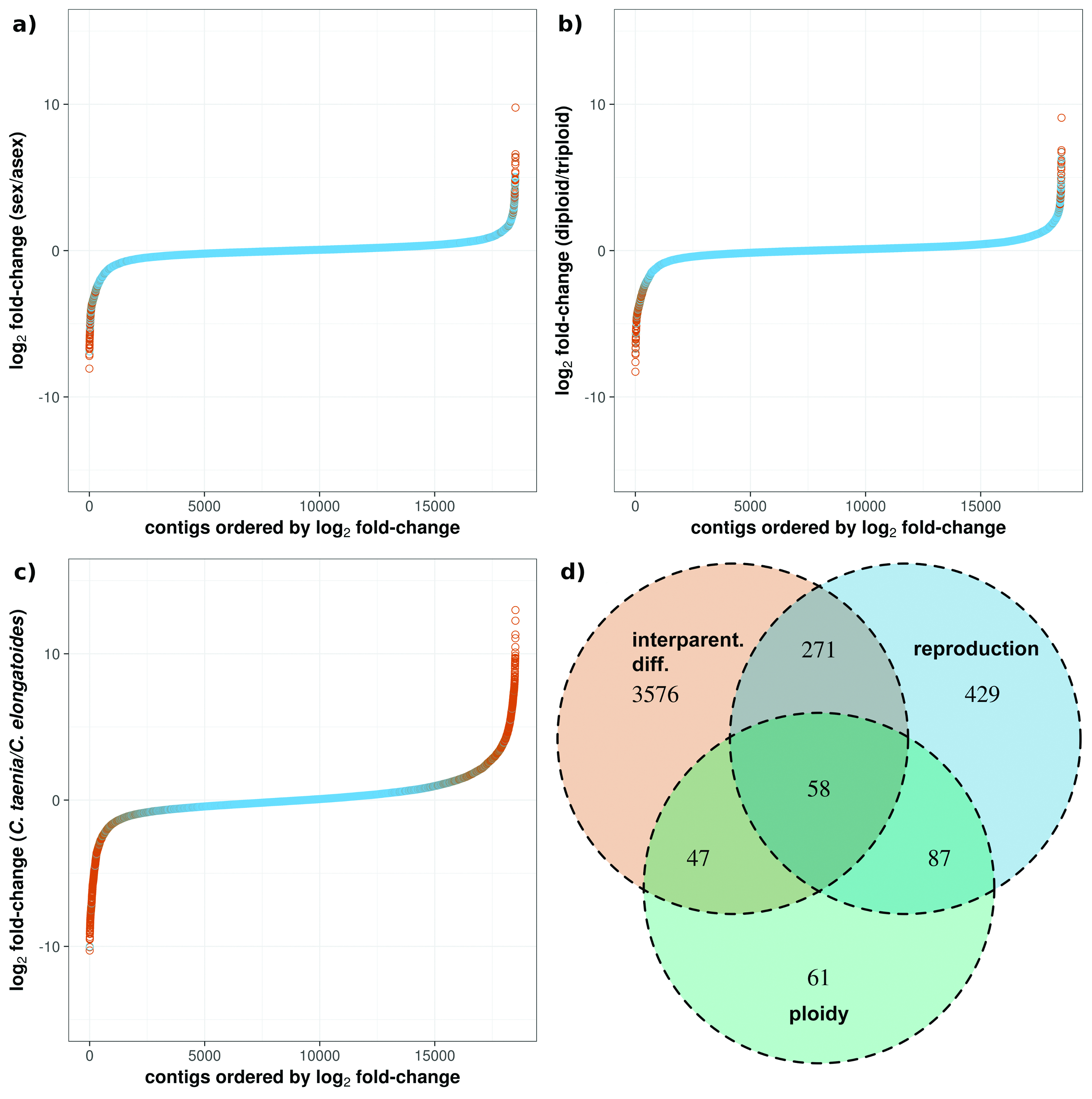

Supplement: msz114_Supplementary_Data [file msz114_supplementary_data.zip › Supplementary_Figure_1.jpg]

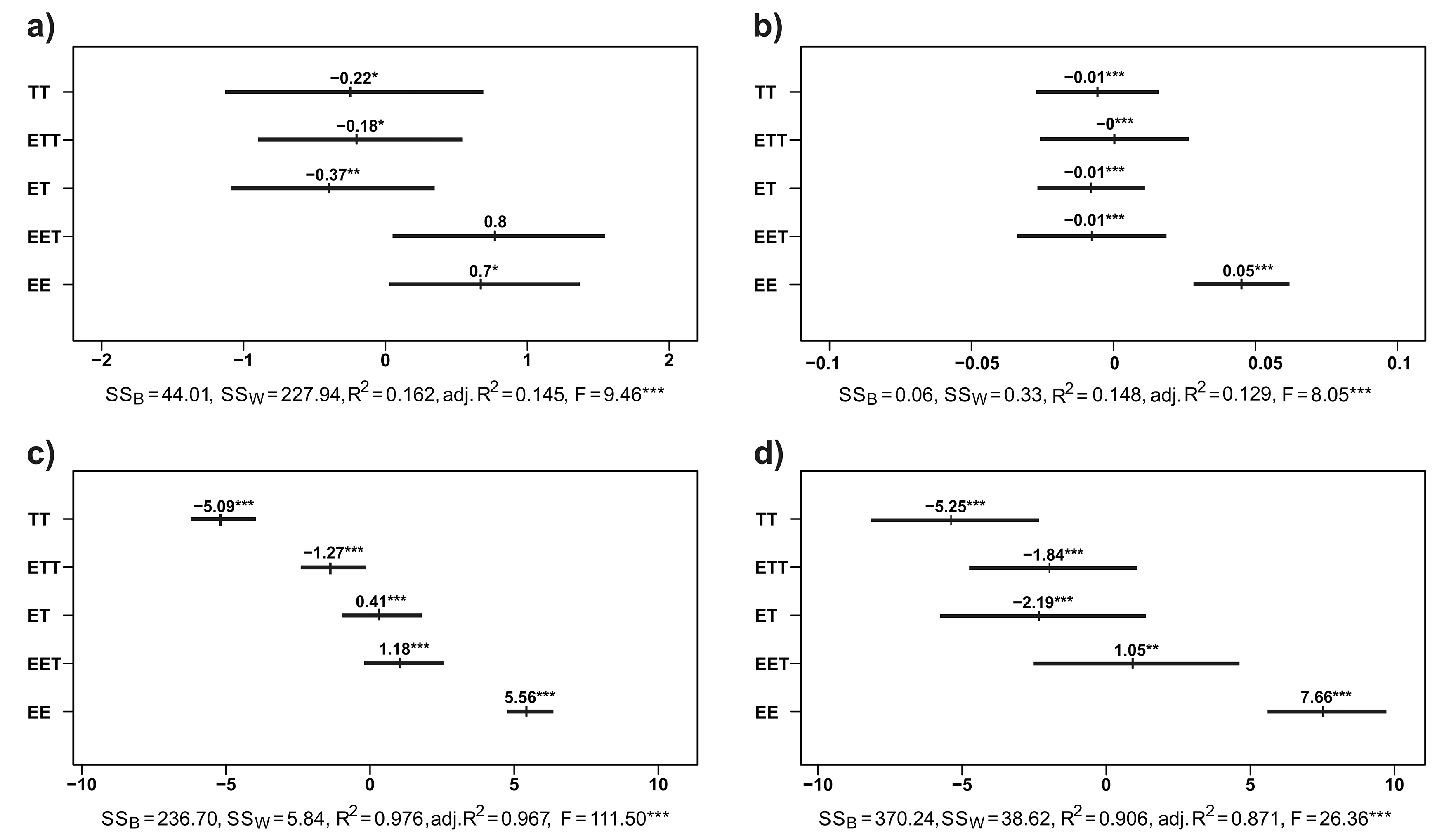

Supplement: msz114_Supplementary_Data [file msz114_supplementary_data.zip › Supplementary_Figure_2.jpg]

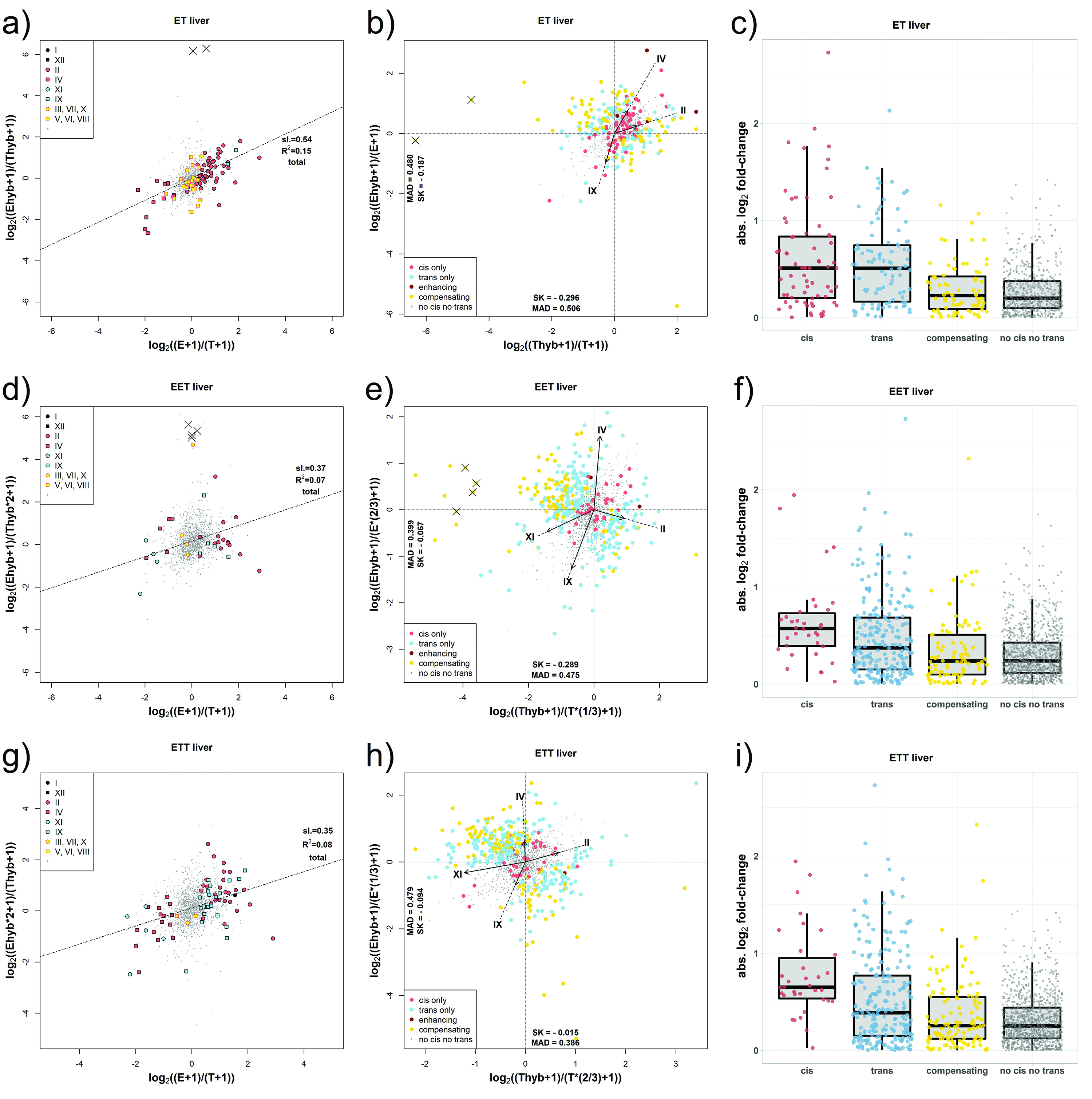

Supplement: msz114_Supplementary_Data [file msz114_supplementary_data.zip › Supplementary_Figure_3.jpg]

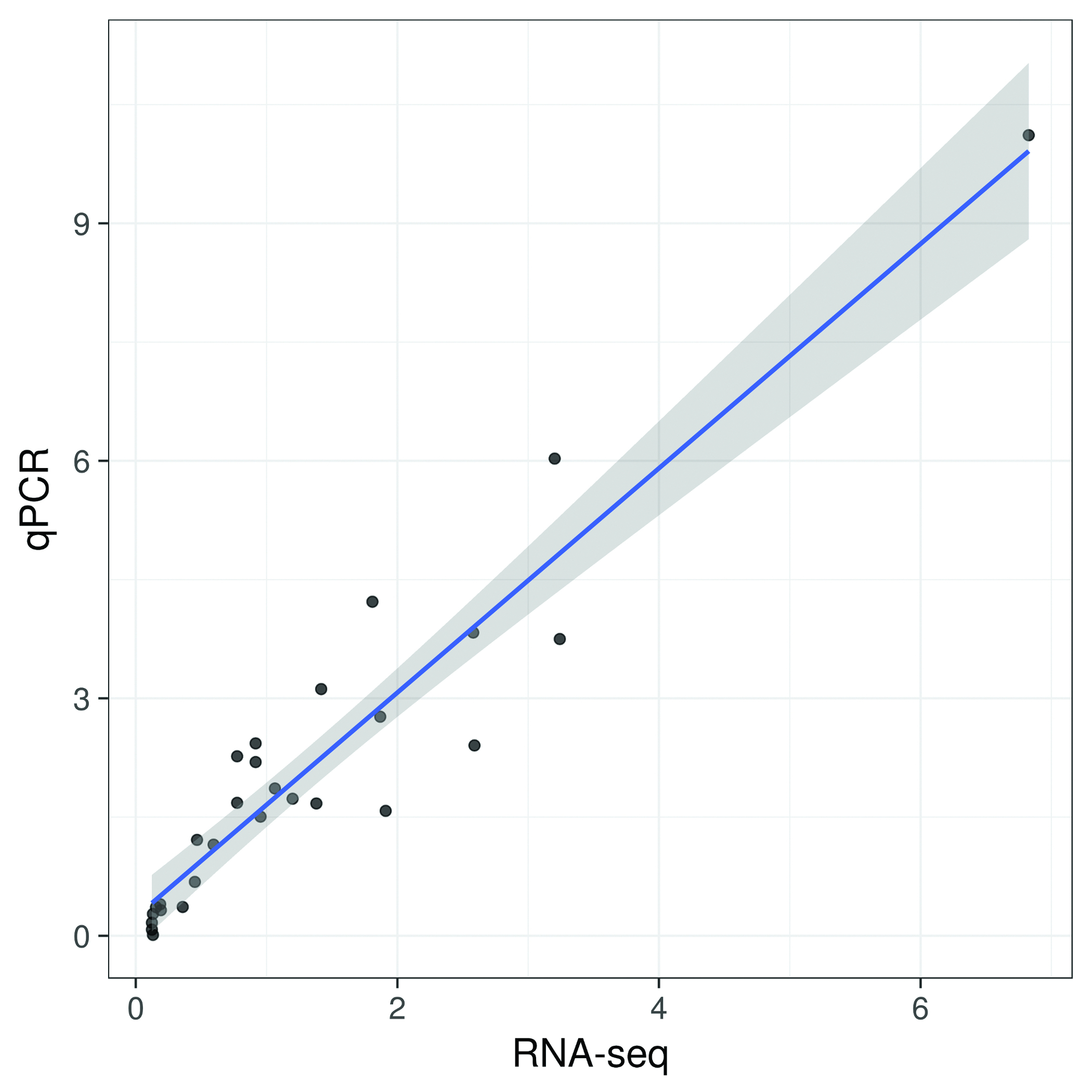

Supplement: msz114_Supplementary_Data [file msz114_supplementary_data.zip › Supplementary_Figure_4.jpg]

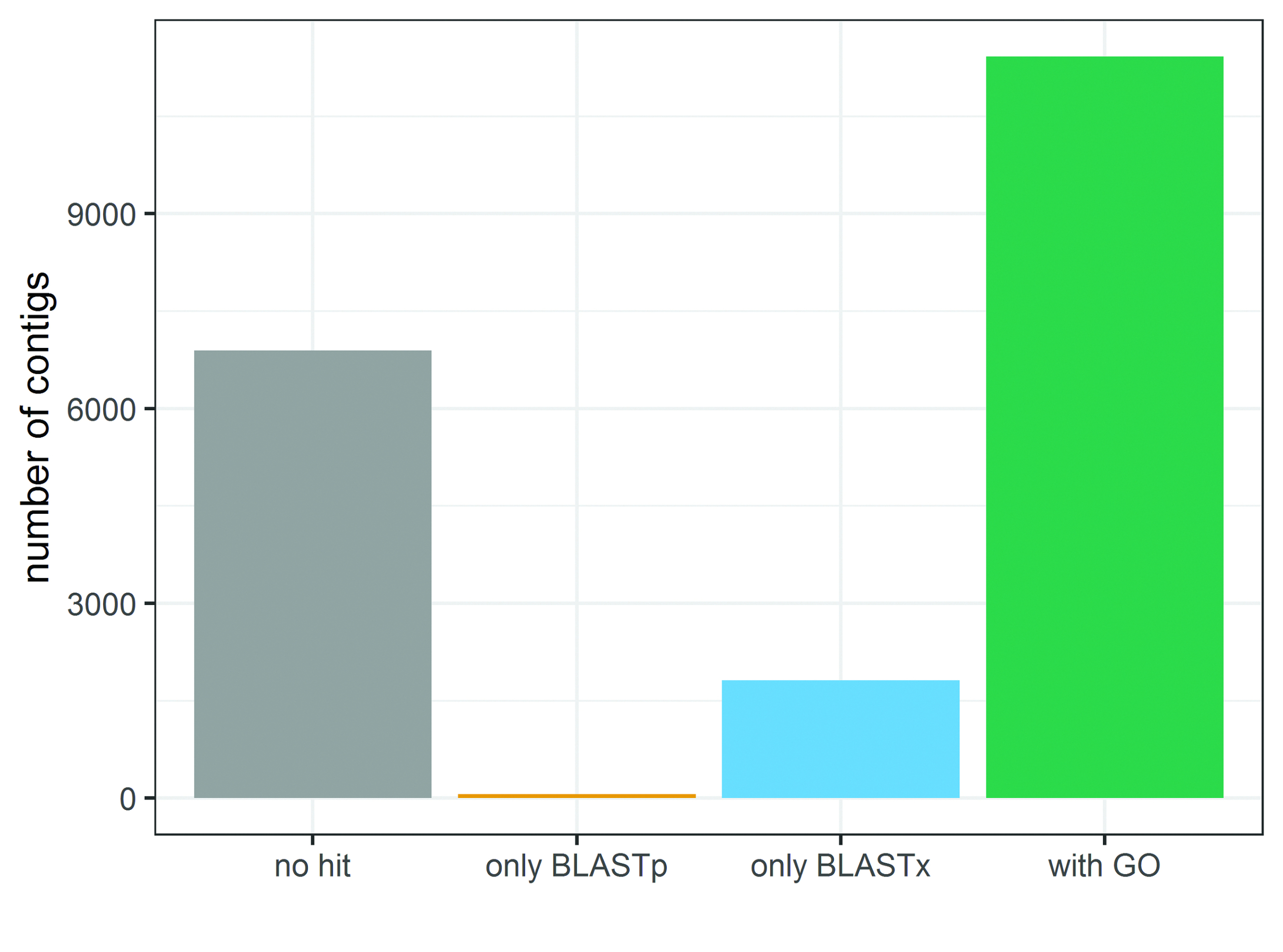

Supplement: msz114_Supplementary_Data [file msz114_supplementary_data.zip › Supplementary_Figure_5.jpg]

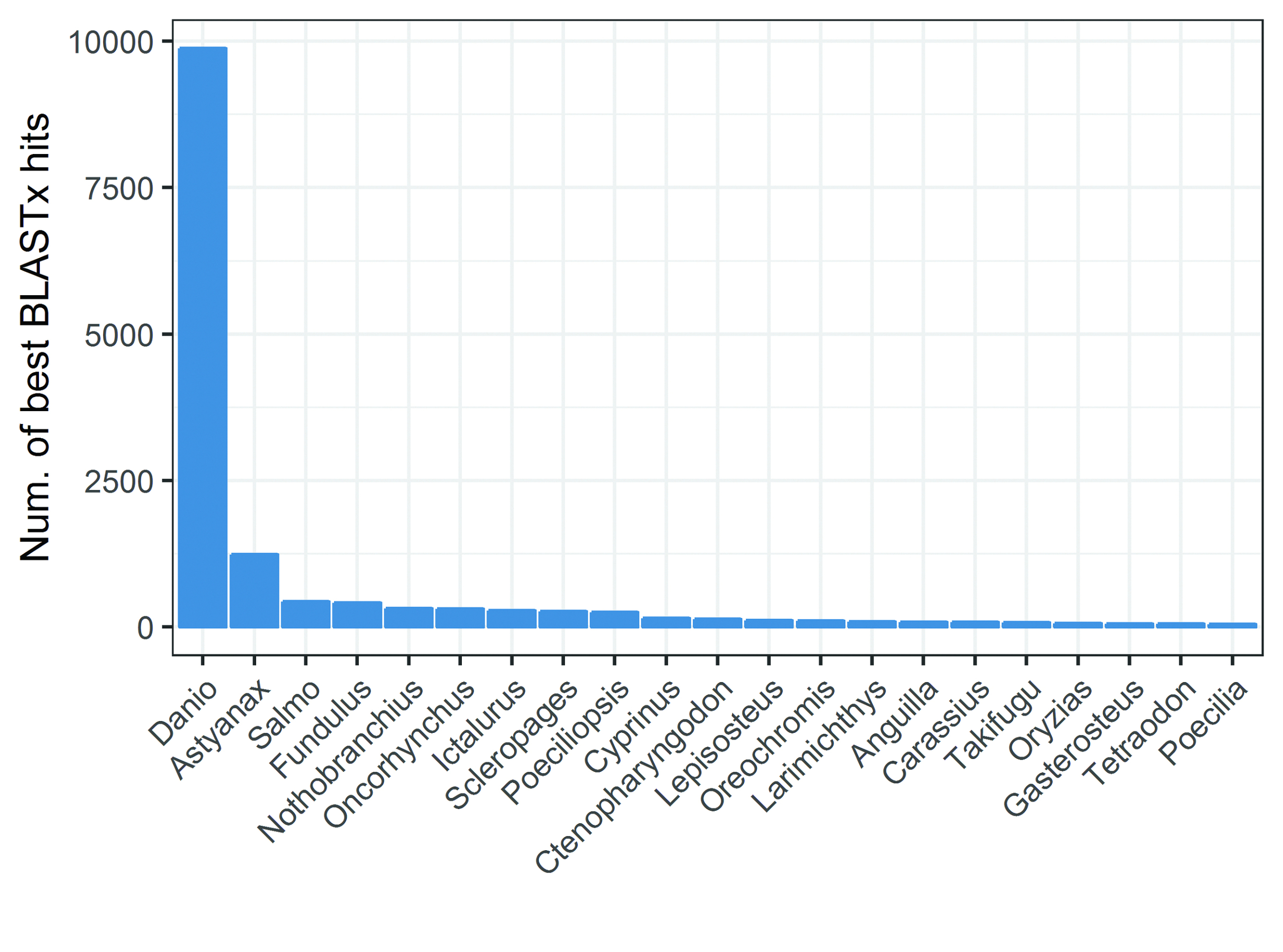

Supplement: msz114_Supplementary_Data [file msz114_supplementary_data.zip › Supplementary_Figure_6.jpg]
